# Modified arthroscopic double row repair of partial thickness tear of the rotator Cuff involving articular and bursal side

**DOI:** 10.4103/0973-6042.41408

**Published:** 2008

**Authors:** Oh Soo Kwon, Jong Hun Ji

**Affiliations:** Department of Orthopaedic Surgery, Daejeon St. Mary's Hospital, The Catholic University of Korea, Daehueng dong, Jung Gu, Daejeon, Korea

**Keywords:** Arthroscopic repair, partial thickness tears, rotator cuff, shoulder

## Abstract

Partial thickness of rotator cuff tears is considered as a common cause of shoulder disability. Various techniques for arthroscopic repair of partial thickness tear of rotator cuff have been reported in the literature. These techniques have addressed the articular side partial thickness cuff tear. We present an arthroscopic repair of partial thickness tear of rotator cuff involving both articular and bursal surfaces without converting into a full thickness tear. Each side of the tear was repaired with suture anchors separately.

## INTRODUCTION

Partial thickness Rotator cuff tears have been recognized as one of the most common pathologies of the shoulder.[1] Fukuda[4] described that these conditions and their characteristics occupy a significant position in the spectrum of rotator cuff disease. Many authors have proposed various techniques for arthroscopic repair of partial thickness tear of the rotator cuff.[5,8,10] Arthroscopic repair techniques of partial rotator cuff tears have mainly focused on the partial articular side tear of the supraspinatus tendon. We present here both articular and bursal side partial thickness rotator cuff tear and we describe the technique of repair for each side of the tear.

## SURGICAL TECHNIQUE

**Figure 1:**
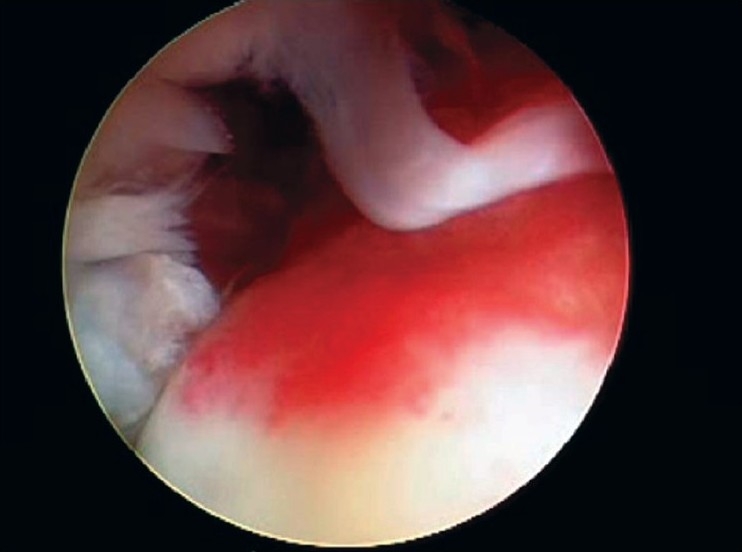
Arthroscopic view showed exposure of footprint due to partial tear at the articular side

**Figure 2:**
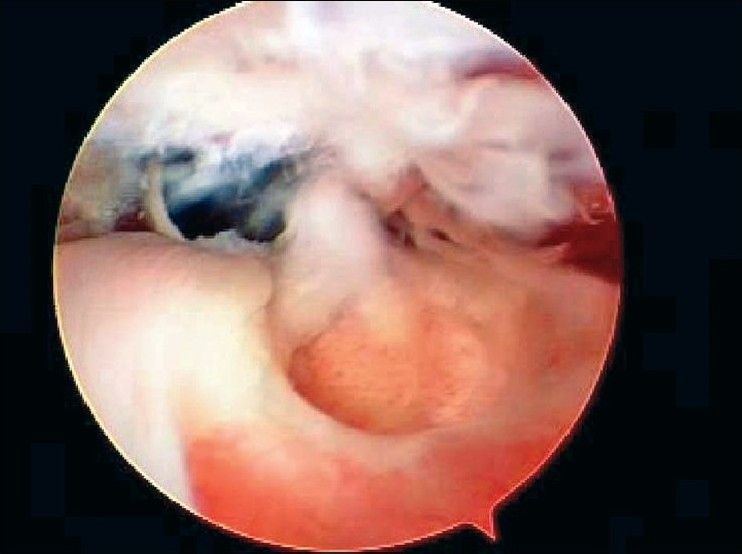
Arthroscopic view revealed bursal side partial tear of the rotator cuff

**Figure 3:**
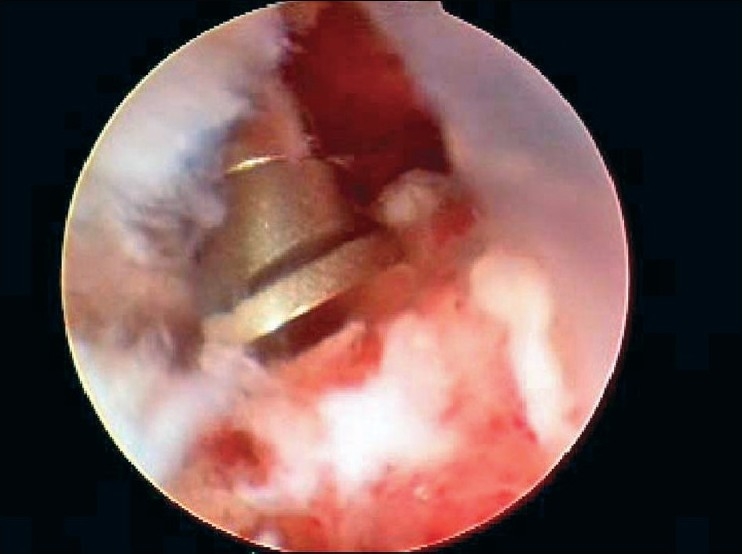
Suture anchor was inserted with transtendon technique

**Figure 4:**
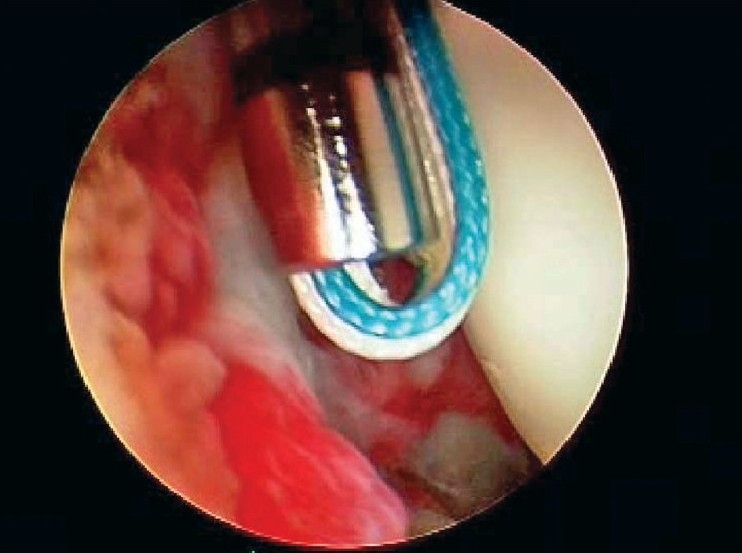
Suture materials were pushed into articular side with using a sheath

**Figure 5:**
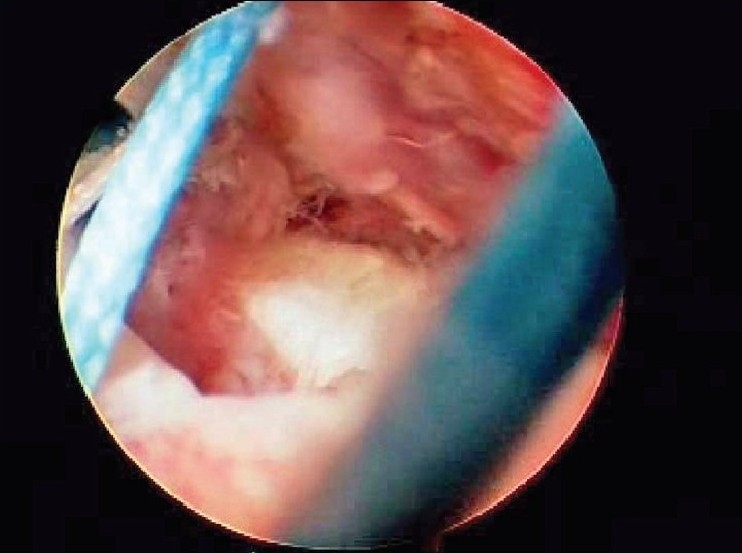
Arthroscopic view demonstrated suture materials brought out into subacromial space

**Figure 6:**
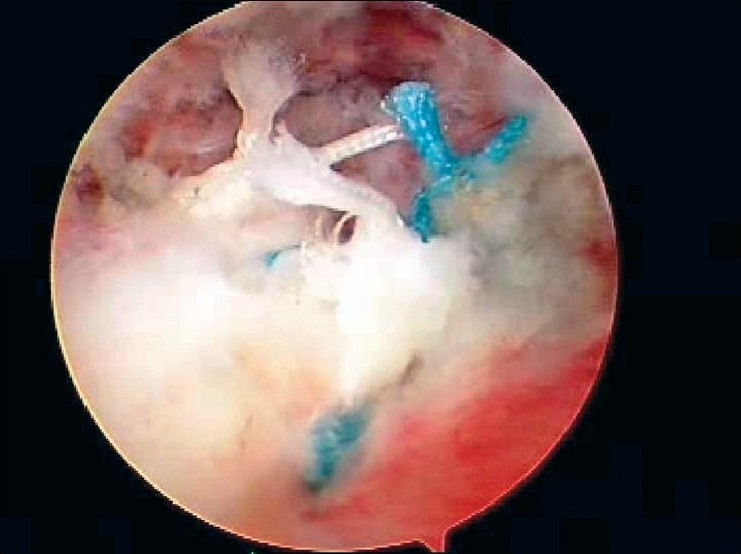
Arthroscopic view presented completely repaired cuff tissue at bursal side

**Figure 7:**
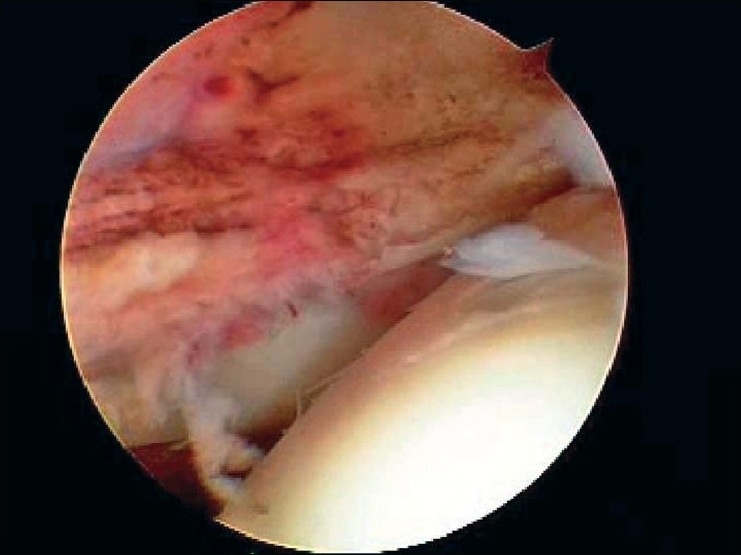
Final check to achieve the restoration of footprint at articular side

**Figure 8:**
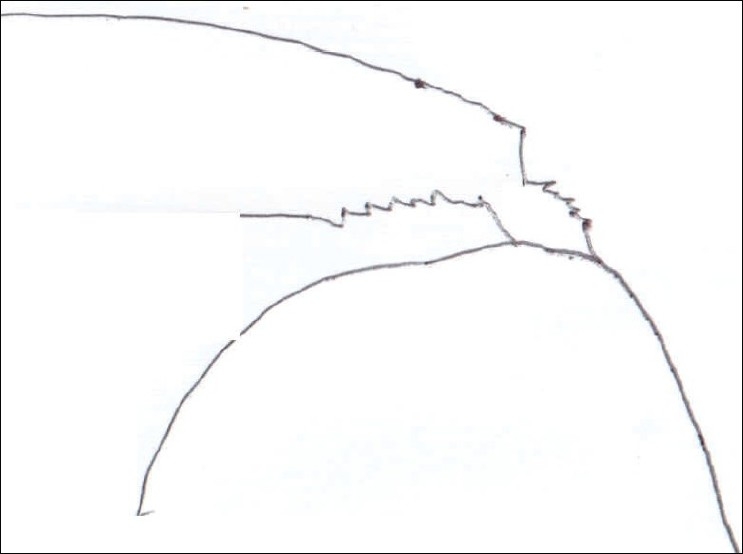
Illustration of partially torn rotator cuff involving both articular and bursal surface

**Figure 9:**
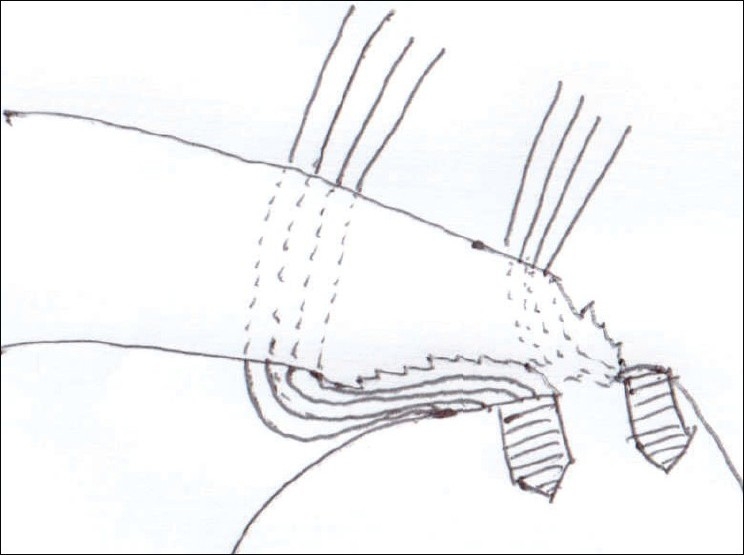
Illustration of suture material passing through each side of the cuff

**Figure 10:**
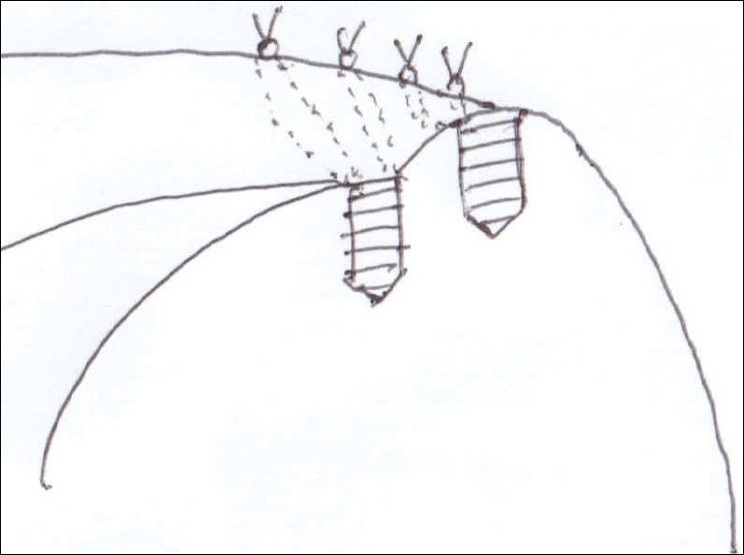
Illustration of complete repair of both sides of tear; horizontal mattress suture for articular side and vertical single suture for bursal side

After induction of general anesthesia, the patient was placed in the lateral decubitus position. 8-10 pounds skin traction was applied with the arm in 30° abduction and 20° forward flexion. Diagnostic glenohumeral arthroscopy was performed using a 30° arthroscope through a standard posterior portal. After confirmation of significant partial thickness articular side rotator cuff tear [Figure 1], two anterior portals were made at rotator interval in an outside-in manner for suture management. The frayed, unstable edge of the tear was debrided with using a full radius resector. After the medial aspect of the footprint was exposed, the size and depth were estimated using a probe notched at 1 mm increments. We used a suture marker to help localize the lesion on the bursal surface. Suture marker (Nylon No. 1) was inserted in to the center of the articular side tear with using a spinal needle percutaneously. Attention was turned to the subacromial space and complete bursectomy was performed to visualize the suture limbs at the time of knot tying, otherwise the sutures limbs could be obscured. A careful evaluation of the bursal surface with an internal or external rotation maneuver was performed to search for any concomitant bursal side tears. After recognizing the dimension of the bursal side tear [Figure 2], the arthroscope was reintroduced into the glenohumeral joint through a posterior portal. An 18-gauge spinal needle was preliminarily inserted from the lateral aspect of the acromion to place the suture anchor (Cork screw, Arthrex, USA). This allowed the anchor to be placed at the medial margin of the rotator cuff footprint just lateral to the articular surface [Figure 3]. Suture anchor was inserted just lateral to the margin of the articular side tear without damaging remaining healthy tissue once spinal needle found proper location for anchor placement. After anchor fixation, double loaded sutures were pushed into the glenohumeral joint with a sheath [Figure 4] and they were pulled out, with using a retriever, into the anterior inferior cannula. One of four suture limbs was withdrawn to the anterior superior cannula without allowing it to be entangled with other suture limbs during suture passage. The medial portion of the tear was pierced with an 18 gauge spinal needle for passing a utility loop (No. 1 nylon) into the anterior superior cannula under the guidance of the marker suture. One limb of each suture that was tagged with the utility loop was brought back into the subacromial space. This suture management was performed strand by strand throughout the entire length of the tear from anterior to posterior at a regular distance. The arthroscope was then put in to subacromial space to check the suture placement at the desired position [Figure 5]. If the suture limb was not placed in the desired position, then the suture limb would be withdrawn into the anterior cannula and the process would be repeated. Before tensioning the suture limbs from the articular side, another suture anchor was inserted for repair of the bursal side tear. Knot tying of sutures from the articular side was followed by those from the bursal side [Figure 6]. The arthroscope was turned to the glenohumeral joint to check the final reduction of articular side tear [Figure 7]. The technique is diagrammatically shown in Figures 8 to 10. Postoperatively, the arm was protected in a sling with an abduction pillow for six weeks. Passive forward exercise began the next day after surgery. Elbow and hand exercise were permitted. At six weeks after surgery, the sling was discontinued and active range of motion exercise was started.

## DISCUSSION

Partial-thickness tears of the rotator cuff are now considered to play a more significant role than was previously thought for inducing disabilities in the shoulder joint.[1,4] Partial thickness rotator cuff tears are usually divided into the articular and bursal side tears according to their location.[1] It is believed that the natural history of the partially torn rotator cuff can result in only limited healing potential and small tears eventually propagate into larger tears.[3,7] Therefore, various surgical interventions have been attempted to repair partial thickness tear of the rotator cuff.[5,8,10] For partial rotator cuff tear involving both the articular and bursal sides, there are no clinical reports regarding repair. However, biomechanical studies have suggested that the insertion regions of both tendon surfaces may be the weakest structurally and may therefore be more prone to failure.[9] Another study demonstrated that a high concentration of stress may occur on both the articular and bursal sides.[2]

Several techniques for repair of partial thickness rotator cuff tear have been reported.[5,8,10] Snyder[7] first described PASTA lesion and transtendon repair technique. Lehman[5] described arthroscopic repair of partial rotator cuff tears by transtendon technique similar to ours. They recommended this technique to preserve remained healthy tissue and promote healing process. Repair after converting to full thickness tear was previously proposed. However, this procedure would not be recommended because conversion to full thickness tear can inadvertently damage the remaining healthy cuff tissues, alter the original footprint and finally create a length-tension mismatch of the repaired cuff.[8] Bey *et al.,*[3] reported that their biomechanical data fails to support the completion and repair of a 50% partial thickness tear. These results suggest repair of only the partially torn cuff and leaving remaining cuff intact. In the case presented in this report, there were substantial robust tissue remaining in the mid-region of the cuff; we believe that this remaining tissue was functional even though biomechanical changes were expected in the tendon. Therefore, it would be better to repair *in situ* rather than completion to full-thickness tear. Although it has both biological and biomechanical advantages, this procedure is technically demanding and time-consuming. This technique requires changing portals between glenohumeral joint and subacromial space up to four times. The arthroscopic pump pressure should be maintained at as low as 50 mmHg to reduce tissue swelling due to extravasation and to help portals placement. Thorough subacromial decompression may be necessary to achieve clear visualization for inspection of the bursal surface and further suture management.

In conclusion, the current technique describes repair of each side of the tear to reconstruct the original footprint without further damage to the remained healthy tissues. Although it is technically demanding, this technique is recommended for the tears involving both the articular and bursal surfaces.
